# A biallelic *MRPL42* variant causes a combined oxidative phosphorylation deficiency syndrome revealed by multi-omics

**DOI:** 10.1038/s41525-026-00564-1

**Published:** 2026-04-03

**Authors:** Felix Boschann, Johannes Kopp, Susanne Römer, Oliver Küchler, Hristiana Lyubenova, Nicolai von Kügelgen, Erik Hertstein, Lea Hagelstein, Christian Becker, Kerstin Becker, Sebastian Brachs, Knut Mai, David Meierhofer, Dominik Seelow, Stefan Mundlos, Denise Horn, Markus Schuelke, Björn Fischer-Zirnsak

**Affiliations:** 1 Institute of Medical Genetics and Human Genetics, Charité – Universitätsmedizin Berlin, corporate member of Freie Universität Berlin and Humboldt Universität zu Berlin, Berlin, Germany; 2 BIH Biomedical Innovation Academy, Clinician Scientist Program, Berlin Institute of Health at Charité - Universitätsmedizin Berlin, Berlin, Germany; 3 FG Development and Disease, Max Planck Institute for Molecular Genetics, Berlin, Germany; 4 Department of Neonatology, Charité-Universitätsmedizin Berlin, Corporate member of Freie Universität Berlin, Humboldt-Universität zu Berlin, Berlin, Germany; 5 Exploratory Diagnostic Sciences, Berlin Institute of Health, Charité – Universitätsmedizin Berlin, Berlin, Germany; 6 Core Unit Bioinformatics (CUBI), Berlin Institute of Health, Charité – Universitätsmedizin Berlin, Berlin, Germany; 7 Cologne Center for Genomics, Medical Faculty, University of Cologne, Cologne, Germany; 8 West German Genome Center, University of Cologne, Cologne, Germany; 9 Department of Endocrinology and Metabolism, European Reference Network on Rare Endocrine Diseases (ENDO-ERN), Charité – Universitätsmedizin Berlin, corporate member of Freie Universität Berlin and Humboldt-Universität zu Berlin, Berlin, Germany; 10 German Centre for Cardiovascular Research, partner site Berlin, Berlin, Germany; 11 Deutsches Zentrum für Diabetesforschung e.V., Geschäftsstelle am Helmholtz-Zentrum München, Neuherberg, Germany; 12 Department of Human Nutrition, German Institute of Human Nutrition, Potsdam-Rehbruecke, Nuthetal, Germany; 13 Berlin Center for Rare Diseases, Charité - Universitätsmedizin Berlin, Berlin, Germany; 14 Mass-Spectrometry Facility, Max Planck Institute for Molecular Genetics, Berlin, Germany; 15 German Center for Child and Adolescent Health (DZKJ), partner site Berlin, Berlin, Germany; 16 Department of Neuropediatrics, Charité-Universitätsmedizin Berlin, Corporate member of Freie Universität Berlin, Humboldt-Universität zu Berlin, Berlin, Germany; 17 NeuroCure Cluster of Excellence, Charité-Universitätsmedizin Berlin, Corporate member of Freie Universität Berlin, Humboldt-Universität zu Berlin, Berlin, Germany

**Keywords:** Biochemistry, Computational biology and bioinformatics, Genetics, Molecular biology

## Abstract

Pathogenic variants affecting components of the mitochondrial translation machinery lead to various impairments of mitochondrial function and thereby cause a spectrum of multisystem diseases. In an infant with a fatal, metabolic multisystem condition we performed a comprehensive multi-omics approach and detected the intronic biallelic variant NM_014050.4:c.219+6 T > A in *MRPL42* (mitochondrial ribosomal protein L42) encoding a component of the large mitochondrial ribosomal subunit. RNA-seq revealed a strong reduction and aberrant splicing of the majority of *MRPL42* transcripts leading to a frameshift and thereby to a premature termination codon: p.(Asn46Leufs*18). However, additional use of the canonical splice site led to a low residual expression of the wildtype transcript and MRPL42 protein abundance was consequently strongly reduced. Complex I and IV activity of the oxidative phosphorylation (OXPHOS) system were reduced and a decrease of complex I, III, IV, and mitoribosomal-related proteins was identified by proteomics. Complementation with wildtype *MRPL42* corrected most of these phenotypes confirming that they were a direct consequence of the limited availability of MRPL42. Our multi-omics data confirm biallelic *MRPL42* loss-of-function as the underlying cause of the fatal mitochondrial disease in our patient. Therefore, we propose MRPL42 deficiency as the cause of a mitochondrial ribosome-related combined OXPHOS-deficiency syndrome.

## Introduction

The mitochondrial ribosome (mitoribosome) is crucial for the translation of 13 mitochondria-encoded proteins serving as structural components of the oxidative phosphorylation system (OXPHOS). The 55S mitoribosome complex within the mitochondrial matrix consists of two multimeric subunits, the small 28S (mtSSU) and the large 39S subunit (mtLSU)^1^. In total, 82 nuclear-encoded mitoribosomal proteins (MRP) and four mitochondria-encoded ribosomal and transfer RNAs (16S rRNA, 12S rRNA, tRNA^Val^, and tRNA^Phe^) are involved in the final 55S assembly in a complex and hierarchically coordinated process^2^. Most MRPs are ubiquitously expressed and have a unique function that cannot be compensated for by other MRPs^3^. To date, pathogenic variants in more than 15 MRP-encoding genes have been associated with defective mitochondrial translation resulting in a highly heterogeneous phenotypic spectrum of autosomal recessive inherited combined OXPHOS-deficiency disorders^4^. The broad clinical spectrum comprises typical manifestations of multisystemic disorders of mitochondrial energy metabolism (i.e., muscular hypotonia, growth retardation, leukodystrophy, hearing loss, cardiomyopathy, myopathy, cognitive impairment, primary ovarian insufficiency, hepatopathy, retinal and renal disease) and ranges from fatal neonatal disease to mild symptoms in adulthood^5^.

The 39S large mitoribosomal subunit is composed of 52 MRPs, the 16S rRNA, and tRNA^Val^^1^. The mtLSU anchors the mitoribosome to the inner mitochondrial membrane within the matrix, stabilizes the mitoribosomal structure, and catalyzes the formation of peptide bonds during translation^6^. One of the 52 MRPs is MRPL42, a small component of the mtLSU with a still largely unknown function. In neonatal mouse cardiomyocytes, knockdown of *Mrpl42* resulted in reduced mitochondrial translation and respiratory function, leading to an exacerbation of ischemia/reperfusion-induced contractile dysfunction and cardiomyocyte apoptosis^7^.

In this report, we show that human MRPL42 deficiency causes fatal neonatal OXPHOS-deficiency (COXPD; OMIM: PS609060) with global respiratory deficiency, sensorineural hearing loss, generalized muscular hypotonia, and lactic acidosis.

## Results

### Clinical description

**Fig. 1:**
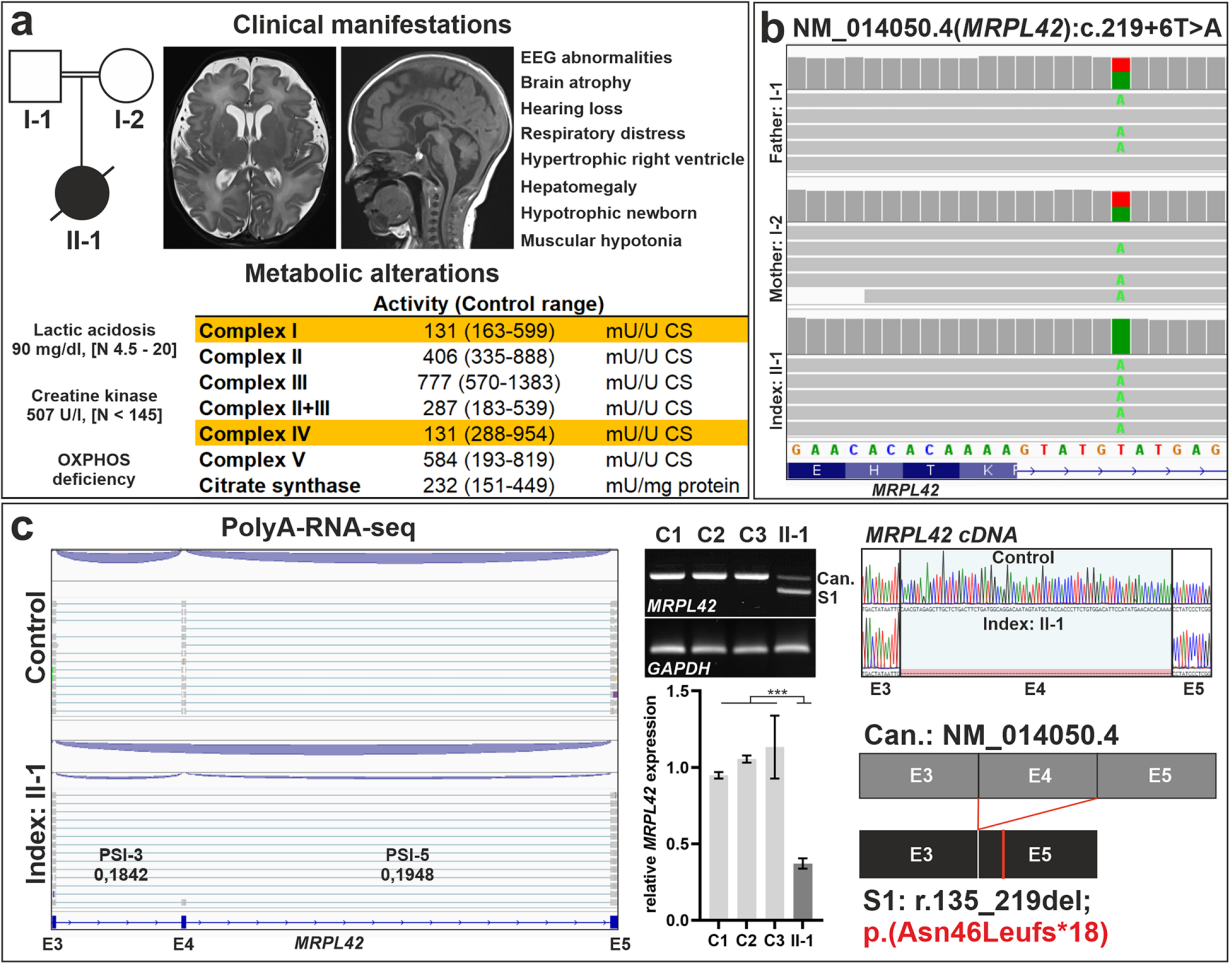
Clinical and genetic characterization of individual II-1. **a** Pedigree of the family from patient II-1. Left panel: T2-weighted cMRI image at postnatal day 3 showing global atrophy of the grey matter. Right panel: T1-weighted image showing atrophy of the *corpus callosum* and the *cerebellum*. Clinical manifestations of the patient II-1 as well as detectable metabolic alterations. Testing of enzyme activities of the respiratory chain revealed a combined deficiency of complexes I and IV in dermal fibroblasts of individual II-1. **b** The variant c.219+6T>A in *MRPL42* (NM_014050.4) was detected in a heterozygous state in both parents (I-1 and I-2) and in a homozygous state in the affected individual II-1. **c** RNA sequencing revealed a predominant aberrant splicing of *MRPL42* exon 4 in II-1 (81% of reads). Usage of PSI matrices showed a usage of the canonical splice site flanking exon 4 in 18% and 19% of the reads respectively^21^. **d** Upper panels: RT-PCR of *MRPL42* exon 3 to 5 showed two products. The upper band represents the canonical spliced transcript (Can.), while the lower band represents the aberrant splice product (S1). Sanger sequencing electropherograms show the exon 4 skipping (r.135_219del). Lower panels: Quantitative RT-PCR showed a strong reduction of *MRPL42* expression. Student’s *t*-test: p*** < 0.001. The schematic overview shows the consequence of these alterations leading to the frameshift p.(Asn46Leufs*18). Diagrams were generated using GraphPad Prism 8.3. The figure was assembled using CoralDRAW 2020.

The female patient was born at 40+3 weeks of gestation with a birth weight of 2600 g (−2.1 SDS), a length of 47 cm (−2.2 SDS) and a head circumference of 33.5 cm (−1.2 SDS) as the first child of consanguineous parents with an APGAR score of 6/6/7. Prenatal ultrasound revealed signs of a cardiomyopathy and a ventricular septal defect (VSD). Prenatal chromosomal analysis and clinical trio exome analysis (restricted to known disease genes) after amniocentesis were unremarkable. Postnatally, the infant had low muscle tone and showed reduced spontaneous movements. She was admitted to the neonatal intensive care unit for respiratory distress and hyperlactatemia (90 mg/dl, [N 4.5–20.0]). Due to frequent apneic episodes and periodic breathing, CO₂ levels rose and invasive ventilation was started at 3 days of age. Two attempts at extubation failed due to lack of spontaneous breathing. Brainstem Evoked Response Audiometry showed evidence of a severe central conduction defect. Cranial magnetic resonance imaging (cMRI) detected global brain atrophy, particularly affecting the grey matter, the *corpus callosum* and the *cerebellum* (Fig. 1a). MR proton spectroscopy revealed mildly elevated lactate levels. Echocardiography showed a hypertrophic right ventricle, a persistent *Foramen ovale* and a small perimembranous VSD. Hepatomegaly was detected by abdominal ultrasound and an electroencephalogram (EEG) showed focal epilepsy-typical potentials.

In the further course, she developed severe metabolic acidosis (pH 7.02, standard base excess −14.7 mmol/L) with hyperglycemia (501 mg/dl, [*N* < 140]), elevated creatine kinase (CK) (507 U/l, [*N* < 145]), and NT-pro BNP (5450 ng/l, [*N* < 125]). She had no active movements of the limbs, presented with persistent muscular hypotonia and areflexia. A neuropathy was suspected due to slowed peripheral nerve conduction velocities. A skin biopsy sample was taken for a fibroblast culture and diagnostic testing of the OXPHOS enzyme activities revealed a combined deficiency of complexes I and IV (Fig. 1a). The family was enrolled in a study on genome sequencing in rare unsolved diseases. Due to progression of symptoms and an unfavorable prognosis with the potential for lifelong mechanical ventilation, life-prolonging measures were discontinued. The patient died at the age of 2 months.

### Disease gene identification

Based on the clinical symptoms described above, we suspected a mitochondriopathy. As the prenatal clinical trio exome analysis was unremarkable, short-read trio genome sequencing and RNA sequencing were conducted on a research basis. First, a virtual panel comprising the Human MitoCarta3.0 listed genes^8^ was used to search for variants in genes implicated in mitochondrial disease. We prioritized rare biallelic variants in the affected neonate with a carrier status in the unaffected parents and a non-benign computational prediction (i.e., CADD v1.7^9^ score >22.7, Revel^10^ score >0.3, SpliceAI^11^ > 0.2). We identified a biallelic intronic variant in *MRPL42*: NC_000012.11:g.93873254T>A, NM_014050.4:c.219+6T>A in the affected individual (Fig. 1b). SpliceAI predicted a weakening of the canonical donor and acceptor splice site of exon 4 (delta scores 0.83 and 0.94, respectively). The database gnomAD v4.1^12^ includes 12 heterozygous carriers of this variant but no homozygous individuals. In addition, no other homozygous *MRPL42* loss-of-function variants are listed in gnomAD. According to the ACMG criteria^13^, we initially classified this variant as a variant of uncertain significance (VUS), since variants in unknown disease genes, i.e., genes of uncertain significance, are per se considered a VUS.

In parallel, the transcriptome data obtained from dermal fibroblasts were analyzed using an in-house tool (SnakeSplice). Differential gene expression was analyzed with Salmon^14^ and DESeq2^15^ was used for fold change estimation. These tools ranked *MRPL42* as the gene with the most significantly reduced expression (Log2FoldChange: −1.6 compared to controls, Fig. S1). Differential splicing analysis using a score of different predictors (including Leafcutter^16^, Fraser 2.0^17^, DexSeq^18^, Whippet^19^, and rMATS^20^) detected an aberrant splicing pattern (exon 4 skipping) in *MRPL42*. Based on Katz et al., quantification of aberrant to wildtype (WT) splicing ratio revealed that exon 4 is skipped in about 81% of the detectable reads (PSI-Metric for exon 3-4: 0.1842 PSI-Metric for exon 4–5: 0.1948^21^, Fig. 1c).

To confirm the findings from GS and RNA-seq analysis, we performed relative quantification of *MRPL42* expression by quantitative PCR (qPCR) and found a strong reduction of *MRPL42* expression in comparison to controls (Fig. 1d). Furthermore, RT-PCR was performed and direct sequencing of the PCR products after gel extraction was conducted, which confirmed the aberrant splicing pattern predicted by SpliceAI and detected in the RNA-seq data. Two bands were detectable, a weaker band corresponding to the size of the reference transcript and an approximately 85 bp shorter band (Fig. 1d). Direct sequencing of this band confirmed the skipping of exon 4 (r.135_219del) (S1, Fig. 1d).

These data show that in the patient’s fibroblasts the detected variant causes a strong reduction of *MRPL42* expression. Splicing of *MRPL42* at the affected position is changed and a frameshift occurs in the majority of detectable transcripts, consequently leading to the generation of a premature termination codon (PTC): NP_751917.1:p.(Asn46Leufs*18). However, in about 20% of the remaining transcripts, the canonical splice site is used and reference *MRPL42* generated (Fig. 1d).

As we suspected this variant to be causal, we aimed to identify additional individuals with homozygous or compound heterozygous variants in *MRPL42*. We used GeneMatcher^22^ and contacted several cooperation partners and reference centers for mitochondriopathies, but we did not identify any other individual carrying suspicious biallelic variants in *MRPL42*. To enable a phenotypic comparison, we summarized the clinical symptoms of all 34 affected individuals with biallelic pathogenic variants in seven MRPL-encoding genes, as identified through a PubMed search^4,23–33^. The phenotypic features occurring in a large fraction of these individuals are structural brain abnormalities, hearing loss, growth delay, failure to thrive, and cardiomyopathy, which are also present in the patient carrying the *MRPL42* variant (Table 1).

**Table 1:** Overview of clinical features of subjects carrying pathogenic variants affecting large mitoribosomal subunits

	Oxidative phosphorylation deficiency syndromes due to:								
	*MRPL42*	*MRPL3*	*MRPL12*	*MRPL24*	*MRPL39*	*MRPL44*	*MRPL49*	*MRPL50*	*MRPLs*
Number of subjects	1	6	1	1	3	7	14	2	35
**Symptoms**									
Sensory-neural hearing loss (SNHL)	1/1	2/6	0/1	0/1	0/3	0/7	9/14	2/2	14/35
Retinopathy	0/1	0/6	0/1	0/1	1/3	1/7	2/14	0/2	4/35
Structural brain abnormalities	1/1	5/6	1/1	1/1	3/3	2/7	8/14	0/2	21/35
Global developmental delay/Intellectual disability	0/1	5/6	1/1	1/1	3/3	2/7	13/14	0/2	25/35
Epilepsy	1/1	0/6	1/1	0/1	2/3	0/7	1/14	0/2	5/35
Respiratory insufficiency	1/1	5/6	1/1	0/1	2/3	0/7	0/14	0/2	9/35
Cardiomyopathy	1/1	6/6	0/1	0/1	2/3	7/7	0/14	2/2	18/35
Growth delay/failure to thrive	1/1	6/6	1/1	0/1	3/3	3/7	0/14	0/2	14/35
Muscular hypotonia	1/1	1/6	1/1	0/1	3/3	1/7	0/14	0/2	7/35
Hepatic abnomalities	1/1	4/6	1/1	0/1	1/3	1/7	0/14	0/2	8/35
Renal abnormalities	0/1	1/6	0/1	0/1	0/3	1/7	1/14	1/2	4/35
Primary ovarian insufficiency	0/1	0/6	0/1	0/1	0/3	0/7	4/14	2/2	6/35
Facial dysmorphism	0/1	0/6	1/1	0/1	0/3	0/7	0/14	0/2	1/35
Age of onset	Neonatal	Neonatal-infantile	Childhood	Infantile	Neonatal-childhood	Neonatal-adolescence	Childhood	Adolescence	–
Age of death	9 weeks	11–15 months	–	–	7–11 months	6–12 months	37 years and unknown in the second individual	–	–
Individuals alive	0/1	3/6	1/1	1/1	1/3	4/7	12/14	2/2	24/35
Metabolic alterations									
Increased serum lactate	1/1	6/6	1/1	1/1	3/3	6/7	14/14	0/2	32/35
Affected OXPHOS complexes	I + IV	I + III + IV + V	I + II + IV	I + IV	I + III + IV + V	I + III + IV + V	I + IV	I + IV	34/35
Mitoribosomal large subunit module	Late-binding	Nascent 16 s rRNA binding	L7/12 stalk	PET-module	A-module	BM1-module	Nascent 16s rRNA binding	BM1-module	–
Molecular genetics									
Reference transcript	NM_014050.4	NM_007208.4	NM_002949.3	NM_145729.3	NM_017446.4	NM_022915.3	NM_004927.4	NM_019051.3	–
Known pathogenic variants	c.219+6T>A; p.(Asn46Leufs*18)	c.49delC; p.(Arg17Aspfs*57)	c.542C>T; p.(Ala181Val)	c.272T>C; p.(Leu91Pro)	c.526delT; p.(Ser176Leufs*8)	c.233G>A; p.(Arg78Gln)	c.125_126delTG; p.(Val42Glyfs*2)	c.335T>A; p.(Val112Asp)	–
		c.950C>G; p.(Pro317Arg)			c.589-924G>A; p.(Gln197Argfs*9)	c.467T>G; p.(Leu156Arg)	c.262C>T; p.(Arg88Cys)		
		255 kb contiguous gene deletion			c.896G>T; p.(Gly299Val)	c.467T>C; p.(Leu156Pro)	c.275A>C; p.(His93Pro)		
					c.921+5G>A; p.(Ile257Hisfs*17),	c.481_484delinsTC; p.(Thr161Serfs*2)			
Biallelic nonsense variants	Yes (residual protein detected)	No	No	No	Yes (residual protein detected)	No	No	No	–
Study PMIDs	This study	21786366, 27815843, 34008913	23603806	32344152	37133451	23315540, 25797485, 34140213, 33742325	40043708	37148394	–

### MRPL42 localization within the mtLSU complex

**Fig. 2:**
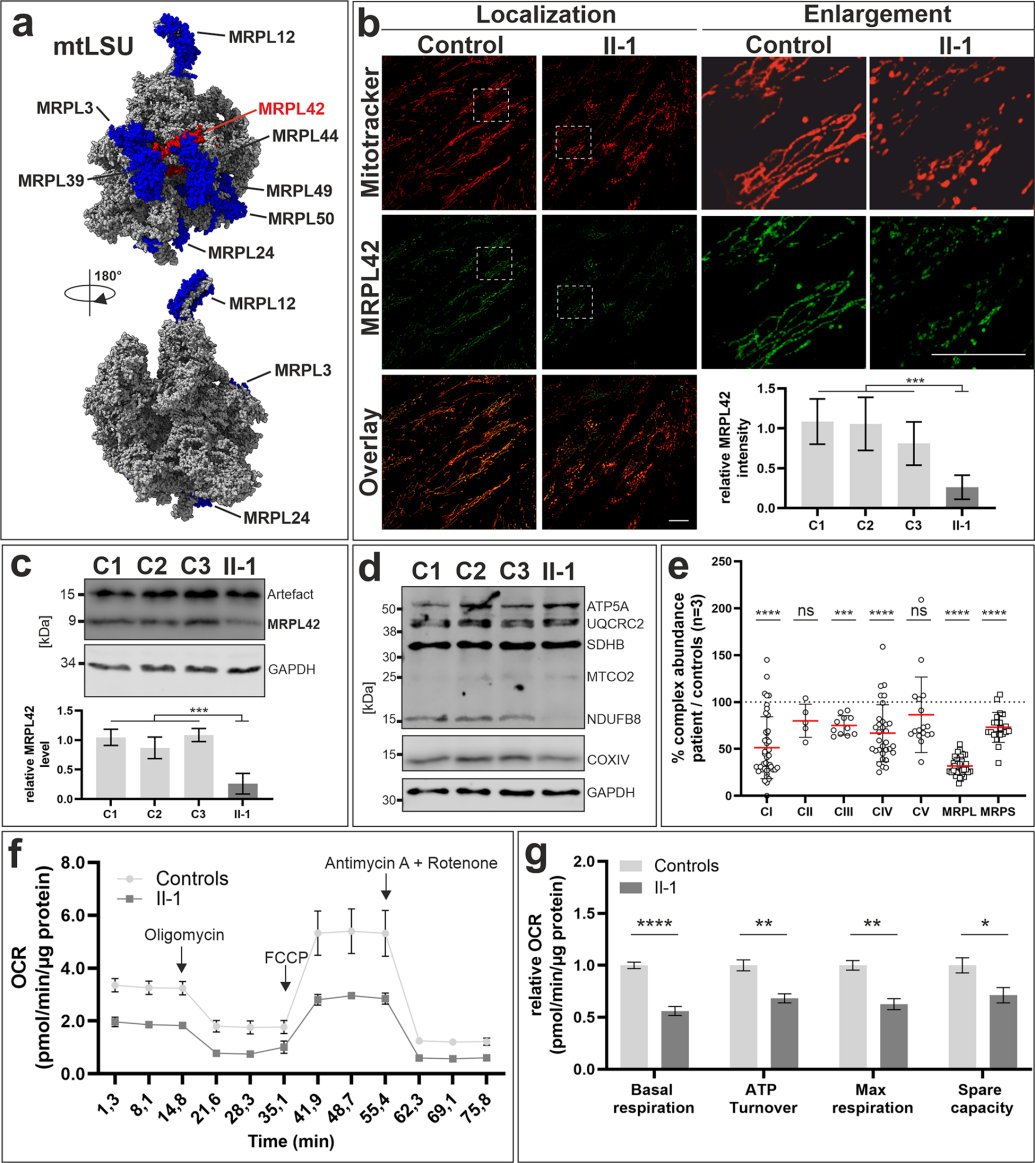
Cellular phenotype and functional consequences. **a** The structure of the mitochondrial 39S large ribosome subunit. Depicted in blue are the so far known components associated with mitoribosome-related combined OXPHOS deficiency syndromes. MRPL42 is depicted in red. MRPL39, MRPL44 and MRPL42 are in close proximity. Images were generated using the ChimeraX Software 1.11. **b** Immunofluorescence staining of Mitotracker (red) and MRPL42 (green) in fibroblasts from individual II-1 and unaffected controls. MRPL42 shows an overlap with Mitotracker in the mitochondria in the control cell line. Fibroblasts of individual II-1 show mild fragmentation of the mitochondrial network. Quantification of fluorescence intensities were measured and revealed a reduction of MRPL42 intensity by 75%. Scale bar: 20 µm. Student’s *t*-test: p*** < 0.001. **c** Immunoblot detection of MRPL42 in lysates of fibroblasts from individual II-1 and unaffected controls (C1-C3). A strong reduction of MRPL42 was detected by quantification of four independent experiments in II-1 compared to controls. Student’s *t*-test: p*** < 0.001. **d** Immunoblot detection of OXPHOS complex components CI-CIV and CV. In II-1, a reduction of the CI and CIV components NDUFB8 and COXIV, respectively, was detected. **e** Proteomics of whole cell protein lysates. Every datapoint represents a detectable protein related to the indicated protein complexes. These analyses revealed reduced abundance of OXPHOS complexes CI, CIII and CIV, while CII remained unchanged. In addition, mitoribosomal complex components compared to three unaffected controls were analyzed and a significant reduction were detectable for MRPL and MRPS related proteins. 2way ANOVA: p*** < 0.001, p**** < 0.0001, ns. not significant. **f**, **g** Analysis of mitochondrial respiration in fibroblasts from individual II-1 and three unaffected controls showing a reduction of oxygen consumption rates (OCR) in all parameters analyzed from II-1. 2way ANOVA: p* < 0.05; p** < 0.01; p**** < 0.0001. Diagrams were generated using GraphPad Prism 8.3. The figure was assembled using CoralDRAW 2020.

In fibroblasts, MRPL42 was found to be co-migrating with the mtLSU complex using complexome profiling^34^. The crystal structure of this complex has been determined using cryo-electron microscopy with a resolution of 3.6 Å^35^. Several entities of combined oxidative phosphorylation deficiency syndromes are caused by biallelic pathogenic variants in genes encoding components of the mtLSU complex. We illustrated the seven thus far known disease-associated proteins and MRPL42 on this mtLSU complex structure bound to the tRNA and found that the localization of MRPL42 is close to several of these components (The closest connection can be found with MRPL39 and MRPL44 (Fig. 2a)).

### Functional consequences of the detected *MRPL42* variant

To investigate the consequence of the identified variant on protein abundance and localization within the mitochondria, we performed immunofluorescence staining in fixed dermal fibroblasts from the affected individual and controls. We analyzed fluorescence intensities from these stainings and detected a strong reduction of MRPL42 protein to 25% in the patient’s fibroblasts compared to the unaffected controls (Fig. 2b). In addition, the staining of the mitochondria using Mitotracker revealed a fragmented mitochondrial network structure in comparison to controls (Fig. 2b). Particle analysis using ImageJ also revealed a slightly increased degree of fragmentation (Fig. S2). To further investigate the observed decrease of MRPL42 in the proband's cells, we performed immunoblotting. In agreement with the immunofluorescence data, protein quantification revealed a strong reduction of MRPL42 abundance (Fig. 2c)

Since MRPL42 is part of the large subunit of the mitoribosome, which is anchored to the inner mitochondrial membrane, immunoblot analysis was performed to investigate a potential impact on the abundance of OXPHOS complex secondary to altered mitochondrial translation. Besides the unchanged abundance of the mitochondrial encoded complex IV subunit MT-CO2, we found a decrease of subunits NDUFB8 (complex I) and COXIV (complex IV), respectively (Figs. 2d and S3).

To investigate whether the decrease of these key components represents a more generalized defect in the patient’s fibroblasts, we performed mass spectrometry-based proteome analyzes from whole cell-lysates in comparison to controls. Complex II components were not altered, but a significant reduction of many complex I and IV subunits was found, while complex III seemed to be only mildly affected (Fig. 2e). Additionally, we analyzed the abundance of mitoribosomal components from the mtLSU and the mtSSU in these data. We found a strong reduction of numerous proteins in both subunits, indicating a general disturbance of mitoribosomal abundance (Fig. 2e). These data provided evidence for a serious impact of the decreased availability of MRPL42 on the mitochondrial ribosomes and the OXPHOS system. Accordingly, we investigated whether these alterations affect the respiration of the proband’s mitochondria. We detected a profound reduction of basal and maximal respiration, sparse capacity and ATP turnover compared to controls (Fig. 2f, g). Together, these data show a severe defect in mitoribosome- and OXPHOS-related processes in the cells from the patient carrying the homozygous *MRPL42* variant NM_014050.4: c.219+6T>A.

### Lentiviral *MRPL42* complementation

**Fig. 3:**
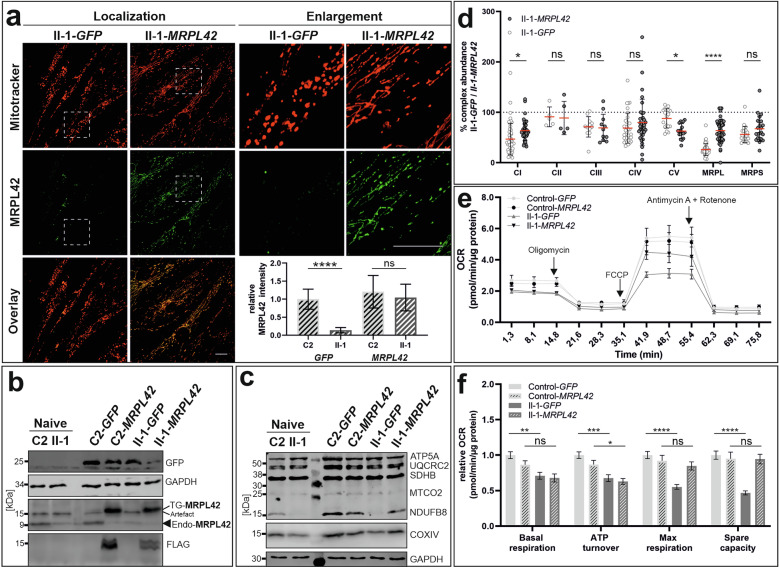
Lentiviral *MRPL42* complementation partially rescues cellular phenotypes. Fibroblasts from individual II-1 and unaffected control C2 were transduced with a lentivirus expressing GFP (II-1-*GFP* and C2-*GFP*) or *MRPL42*-ORF (II-1-*MRPL42* and C2-*MRPL42*), respectively. **a** Staining of Mitotracker (red) and immunolabeling of MRPL42 (green) in fibroblasts from individual II-1 after overexpression of GFP and MRPL42. Overexpression of *MRPL42* in II-1-*MRPL42* restores the mitochondrial network integrity partially Scale bar: 20 µm. Relative expression of MRPL42 was quantified. This analysis shows a restoration in the cells from patient II-1. **b** Immunoblot detection of GFP and MRPL42 showing the effective expression of the transduced proteins. Labeling of FLAG-tag and MRPL42 showed an overexpression of MRPL42. **c** OXPHOS complex components related to CI-CV after lentiviral overexpression of *GFP* and *MRPL42*. OXPHOS complex components NDUFB8 and COXIV were normalized upon MRPL42 complementation in II-1-*MRPL42* compared to C2-*MRPL42*. **d** Proteomics of OXPHOS complex CI-CV and mitoribosome related proteins. Every datapoint represents a detectable protein related to the indicated protein complexes. A normalization of OXPHOS complex components related to CI and CIV was observed after *MRPL42* overexpression. In addition, a normalization of MRPL and MRPS component abundance was found after MRPL42 complementation. II-1-*GFP* was normalized to C2-*GFP*, II-1-*MRPL42* was normalized to C2-*MRPL42*. **e**, **f** Analysis of mitochondrial respiration using Seahorse. *MRPL42* complementation results in slight increase of basal respiration and ATP turnover whereas a normalization of maximal respiration and spare capacity were detected in II-1-*MRPL42* cells compared to C2-*MRPL42* cells. 2way ANOVA: p^*^<0.05; p**<0.01; p^***^<0.001; p^****^<0.0001; ns. not significant. Diagrams were generated using GraphPad Prism 8.3. The figure was assembled using CoralDRAW 2020.

To examine whether the observed phenotypes in the patients’ fibroblasts can be explained by the MRPL42 deficiency, we performed a lentiviral-based gene transfer to complement the patient’s cells with the wild-type form of MRPL42 (Fig. S4). The successful gene transfer was confirmed by quantitative RT-PCR showing a 150% *MRPL42* overexpression in transduced control and proband’s fibroblasts (Fig. S5). Immunofluorescence staining confirmed strongly increased MRPL42 abundance within the cells, while transduction with an empty green fluorescent protein (GFP)-linked vector had no effect (Fig. 3a). Furthermore, the mitochondrial network visualized using Mitotracker appeared more tubular compared to GFP-transduced cells. The objectivation via ImageJ revealed a partial restoration of this mild alteration on the mitochondrial network integrity (Figs. 3a and S6). Immunoblot analysis showed a strong overexpression of normal *MPRL42* fused to a FLAG-tag (TG: 15 kDa) in the transduced control and patient’s cells, which most probably led to a suppression of endogenous MRPL42 (Endo: 9 kDa) (Fig. 3b). In transduced patient’s cells (II-1-*MRPL42*), the abundance of complex subunits NDUFB8 (complex I) and COXIV (complex IV) returned to normal levels, while cells transduced with an empty GFP-linked vector (II-1-*GFP*) resembled the naive cells. For note the abundance of the mitochondrial encoded complex IV component MT-CO2 (COXII) remained unchanged in all conditions and comparable to the levels detected in the native cells (Fig. 3c).

To investigate whether the complementation reversed the observed changes of mitoribosome and OXPHOS component abundance, we performed mass spectrometry-based proteome analysis. Compared to the cells transduced with the control vector (*GFP)*, the abundance of complex II remained unchanged in both conditions as expected. Changes in complex I and IV abundances persisted in the patient cells lentiviral transduced with GFP (II-1-*GFP*) but were slightly corrected in the patient’s cells transduced with WT *MRPL42 (*II-1-*MRPL42)*. Surprisingly, the abundance of ATP synthase proteins was reduced in the patient’s cells transduced with WT *MRPL42 (*II-1-*MRPL42)* (Fig. 3d). The analysis of components of the mtSSU and mtLSU in these cells also revealed a strong correction in the transgene transduced cells II-1-*MRPL42* (Fig. 3d). These results show that the complementation with normal MRPL42 is sufficient to partially correct the observed mitoribosome and OXPHOS complex alterations.

Additionally, we examined whether this partial compensation also affected the OXPHOS system function. Therefore, we measured mitochondrial respiration in the proband’s fibroblasts lentiviral transduced with either the *MRPL42 (*II-1-*MRPL42)* or GFP (II-1-*GFP)* in comparison to a control cell line transduced with the same constructs *(*Control-*MRPL42)* or GFP (Control-*GFP)*. Indeed, we observed an improvement in basal respiration and ATP turnover after MRPL42 complementation. Strikingly, maximal respiration and the sparse capacity, measured after uncoupling with FCCP, returned to almost completely normal levels in the patients' cells complemented with MRPL42 (Fig. 3e, f).

In summary, we detected an intronic variant in *MRPL42*(NM_014050.4):c.219+6T>A, NP_751917.1:p.(Asn46Leufs*18) and found strong alterations of the mitoribosome and the OXPHOS system using a comprehensive multi-omics approach in the proband fibroblasts. In addition, we were able to rescue the observed defects in the patient’s cells by wild-type *MRPL42* transduction. As a consequence of this extensive functional characterization and in analogy to other mitoribosome-related diseases, which are caused by biallelic loss-of-function variants, we propose a pathogenic classification: PVS1_str(RNA), PM2, PM3_sup, PP4 and submitted this information to the ClinVar database (Variation ID: 4071942). Further cases are required to verify the assumed mode of inheritance and pathomechanism.

## Discussion

In this study, we identified a homozygous intronic variant in *MRPL42* leading to aberrant splicing in a neonate presenting with a severe mitochondriopathy. Notably, the intronic variant is detectable in principle by exome sequencing, but had not been reported in the prenatal setting, because *MRPL42* was not a known disease gene. Nevertheless, only the combination of RNA sequencing and mass spectrometry in a multi-omics approach provided a comprehensive and important dataset for interpreting the functional consequences of the detected variant.

So far, pathogenic variants in seven other genes encoding different proteins of the mtLSU have been reported in a total of 34 individuals^4,23–33^. A comparison of these cases showed that the clinical symptoms and disease severity of the affected individuals are heterogeneous; however, they often share phenotypic features such as failure to thrive, growth delay, cardiomyopathy, sensorineural hearing loss, neurodevelopmental delay/intellectual disability, structural brain abnormalities, muscular symptoms such as hypotonia or weakness, respiratory distress, and lactic acidosis. Overall, the symptoms of the affected individual carrying the *MRPL42* pathogenic variant are in line with the known phenotypic spectrum of the previously published mtLSU-associated mitoribosomal diseases. Cases with milder symptoms occurring in early adulthood, such as exercise intolerance or primary ovarian insufficiency, have been described and were predominantly associated with homozygous missense variants^4,23^. In most cases with an early lethal course, a PTC variant was detected on at least one allele. However, in all such cases, a certain amount of functional protein is formed, for example, by the partial usage of canonical splice sites^24^. This emphasizes the importance to further examining splice site variants by RNA analysis to avoid reporting even more variants of unclear significance. Our functional analyzes in fibroblasts revealed a strong degradation of the aberrant *MRPL42* transcripts, while in the remaining fraction of molecules, the canonical splice site was used, resulting in some residual and functional wild-type MRPL42. Notably, it is possible that the degree of degradation and the ratio of canonical to aberrant splicing may be different in other tissues and thus the amount of functional MRPL42 protein may vary. However, a low level of residual activity is consistent with other *MRPL* variants, detected in cases with a severe neonatal course of the disease^24^. To our knowledge, a complete loss of a MRPL component has not yet been described, as this would presumably be embryonically lethal. This is further underlined by the fact that all so far published *Mrpl* knockout models are non-viable^3^. Consequently, we speculate that this residual availability of functional MRPL42 might be an explanation for the survival of the individual during the prenatal and early neonatal phase.

Interestingly, in murine neonatal cardiomyocytes, *Mrpl42* was found to be the most significantly downregulated gene after myocardial ischemia. This work also showed that siRNA-mediated knockdown of *Mrpl42* led to impaired mitochondrial translational efficiency and respiratory function, causing exacerbation of cardiac contractile function and apoptosis^7^. Likewise, the splice variant in *MRPL42* results in a strong reduction of functional MRPL42 protein, leading to impaired mitoribosome stability and severe OXPHOS dysfunction. A combined OXPHOS deficiency was also found in previously published cases, with the individual complexes affected to varying degrees depending on the tissue examined, often more pronounced in cardiac muscle and skeletal muscle than in fibroblasts. In most cases, there was at least a deficiency of complexes I, IV and sometimes III, while complex II remained unaffected, which is a clear indicator of a mitochondrial translation defect. The alterations in the fibroblasts from patient II-1, detected by complex activity and proteomic measurement, are in line with these observations but no direct proof of a translational defect. Interestingly, our data show that the mitochondrial encoded component of complex IV, MT-CO2 (COXII), is not altered in any of the analyzed conditions. This is not a unique finding. In patient fibroblasts with pathogenic variants in *MRPL44* a decreased abundance of COXII was found; however, no defect in de novo mitochondrial translation^32^. Furthermore, in fibroblasts from individuals with *MRPL49* pathogenetic variants, no effect on the abundance of COXII was detectable^4^. The protein levels detected in our whole cell proteomic approach represent the situation at the time of lysis. However, the abundance of proteins depends on the equilibrium between synthesis and degradation. Many mammalian mitochondrial proteins, including COXII, have a relatively long half-life^36^. Consequently, these proteins have a low turnover. Therefore, we speculate that even a small amount of functional mitoribosomes is still sufficient to translate COXII to an extent that remains relatively constant due to its long half-life. Further studies, including a full inactivation of *MRPL42* and other MRPL components using pulse chase experiments with labeled amino acids, are needed to resolve this phenomenon.

Furthermore, we observed a strong reduction of a large fraction of components from the mtLSU and the mtSSU. The latter might be secondary due to the reduction of the large subunit, which is crucial for membrane localization and stabilization of the complete mitoribosome^6^. This implies, however, at least an altered translation activity within the mitochondria. Since this would influence mitochondrial ATP synthesis, we analyzed oxygen consumption rate (OCR) measurements that showed impaired respiratory function in the proband’s fibroblasts. The lentiviral complementation revealed a partial to complete rescue of these changes. Interestingly, the rescue was less efficient for the basal respiration and ATP turnover than for the maximal respiration and sparse capacity, which could be explained by the culture conditions. Under standard conditions, the cells generate energy preferentially via glycolysis. For the respiratory measurements, the cells were starved with reduced glucose levels. We speculate that the less efficient rescue for basal respiration and ATP turnover can be explained by a reduced dependency on oxidative phosphorylation even under these conditions. This is further supported by the almost completely rescued maximal respiration and sparse capacity. These values were obtained after uncoupling and are therefore independent from glycolysis. In this regard, also some of the mild changes within our proteomics data could be explained and further interpreted, such as the unclear reaction of complex V after MRPL42 transduction. Therefore, a complete replacement of glucose by galactose could lead to a more uniform rescue. However, the culture conditions need to be extensively tested because the cells would very likely not survive the cultivation under these stressful conditions.

Our data indicate that MRPL42 deficiency is the cause of the observed cellular phenotypes. These findings were consistent with the rescue studies conducted in murine cardiomyocytes^7^. In contrast to the other disease-associated proteins of the large subunit, MRPL42 was found to be a late-binding protein, not present in the key mtLSU assembly modules or central domains^2^. The exact function of the relatively small MRPL42 protein within the mtLSU remains unknown, but it was hypothesized to stabilize bypass segments of the mtLSU^6^. While we found a strong downregulation of *MRPL42* on transcript level, no other genes related to the mitoribosomal or OXPHOS function were affected. However, on the protein level, we observed strong changes in both processes that normalized after MRPL42 complementation. In agreement with the abovementioned functional hypothesis, our results imply that MRPL42 is relevant for stabilizing the mitoribosome.

In summary, our data show that the identified biallelic variant causes a strong but not complete MRPL42 deficiency. Our proteomic data not only helped during the variant interpretation process but also provided further evidence that MRPL42, although not essential for mitoribosomal assembly, is important for overall stability of the mitoribosome. The comprehensive multi-omics approach, including genome sequencing, RNA sequencing, and mass spectrometry-based proteomics and the lentiviral rescue of the variant’s functional consequences ultimately led to the conclusion that the detected variant in *MRPL42* can be regarded as the cause of a mitoribosome-related combined OXPHOS deficiency syndrome.

## Methods

### Trio genome sequencing

The study was performed in accordance with the Declaration of Helsinki protocols and approved by the Ethics Committee the Charité—Unversitätsmedizin Berlin (approval EA2/205/21). The parents gave their written consent for all clinical and molecular studies in this report. In addition, the parents gave their written consent for the publication of the molecular details as well as images. We sampled peripheral blood from the affected individual and her unaffected parents and extracted the DNA according to standard procedures. Genome sequencing (GS) was performed using our established pipeline^37^. The reads were mapped to version GRCh37 (UCSC hg19) with the BWA MEM2 tool (Burrows–Wheeler Aligner^38^). Single-nucleotide variants and short indels were called with the Genome Analysis Toolkit (GATK) in haplotype caller mode aligned to the GATK Best Practices^39,40^. We used Mehari (publication in preparation) for variant annotation, and Varfish for filtering and further data analysis as described previously^37,41^.

### Cell culture

Dermal fibroblasts from the patient and unaffected unrelated controls were cultivated in DMEM (4.5 g/l Glucose, Gibco, ThermoFisher) with 10% fetal calf serum (Gibco, ThermoFisher), 1% GlutaMAX (Gibco, ThermoFisher) and 1% penicillin/streptomycin (Gibco, ThermoFisher) at 37 °C and 5% CO_2_.

### RNA extraction and cDNA synthesis

Confluent dermal fibroblasts were seeded at 2 × 10^5^ cells/well in a 6-well plate, cultured for 72 h and washed twice with phosphate-buffered saline (PBS) before lysing. Cells were lysed in Trizol (Thermo Fisher Scientific) and total RNA was extracted using Phenol/Chloroform extraction. cDNA was transcribed using the RevertAid H Minus First Strand cDNA Synthesis Kit (Thermo Fisher Scientific).

### RNA sequencing

We performed a poly-A enrichment from total RNA preparations. Libraries and sequencing were performed as previously described^37^. 60–70 million paired-end sequence reads were generated per sample. For visualization purposes, RNA-Seq reads were mapped to the human genome (GRCh37, Ensembl release 110) using STAR version 2.7.11a^42^. Gene expression quantification for downstream analyzes was performed using Salmon^14^ based on transcript-level abundance estimates using the GRCh38 reference (Ensembl release 110).

### Qualitative RT-PCR and Sanger sequencing

Primers located in exons 3 and 5 of *MRPL42* were used for cDNA amplification with FIREPol Mastermix (Solis BioDyne) in a ProFlex PCR System (Thermo Fisher Scientific) and subsequent agarose gel electrophoresis. After gel extraction of fragments using QIAquick Gel Extraction Kit (Qiagen), Sanger sequencing was carried out using BigDye Terminator v3.1 Cycle Sequencing Kit (Thermo Fisher Scientific) on an ABI 3500×L DNA Analyzer (Thermo Fisher Scientific). Primer sequences are listed in Table S1.

### Quantitative RT-PCR

Quantitative RT-PCR was carried out using Eva Green (Solis BioDyne) on a QuantStudio 3 system (Thermo Fisher Scientific) using two different pairs of primers located in exon 2-3 and 5-6. All primer sequences are listed in Table S1.

### MRPL42 localization within the mtLSU complex

To visualize the MRPL42 protein in the context of the large complex of the mitochondrial ribosome, we used the cryo-electron microscopy-generated crystal data from Saurer et al.^35^. We used the ChimeraX Software 1.11^43^ and visualized the 39S human mitochondrial large ribosomal subunit (https://www.rcsb.org/structure/8OIT). The complex is presented in grey, the components linked to mitochondrial ribosome-related combined OXPHOS-deficiency syndromes, MRPL3, MRPL12, MRPL24, MRPL39, MRPL44, MRPL49 and MRPL50^4,23–33^ in blue. MRPL42 is depicted in red.

### Immunofluorescence

Dermal fibroblasts were grown on glass coverslips for 72 h and were fixed for 10 min in cold 100% methanol (Carl Roth) at 4 °C followed by blocking in 3% bovine serum albumin (BSA) in 1× PBS for 20 min. Immunofluorescence staining was performed for MRPL42 (rabbit anti-MRPL42; #17300-1-AP, Proteintech 1:500) overnight in 3% BSA in 1x PBS and secondary anti-rabbit IgG Alexa Fluor 488 antibody (#A21206, Invitrogen 1:500) for 1 h. Cells were incubated with Mitotracker Deep Red (#8778, Cell Signaling Technology) at a final concentration of 0.5 µM for 30 min at 37 °C, fixed for 15 min at −20 °C with 100% methanol (Carl Roth), and mounted in Fluoromount G (Biozol). Images were taken using a LSM700 (Zeiss). Each experiment was performed three times. For mitochondrial fragmentation studies, images of MRPL42 stainings were analyzed with ImageJ. Individual cells were selected and analyzed with the “Analyze Skeleton (2D/3D)” function. For each cell, the number of structural endpoints was normalized to the area of the cell and displayed relative to the mean value of controls.

### Proteome analysis

Confluent dermal fibroblasts were seeded at 2 × 10^5^ cells/well in a 6-well plate, cultured for 48 h and washed twice with PBS before lysing. The human fibroblast samples were lysed under denaturing conditions in 100 µL of a buffer containing 3 M guanidinium chloride (GdmCl), 10 mM tris(2-carboxyethyl)phosphine (TCEP), 40 mM chloroacetamide and 100 mM Tris-HCl pH 8.5. Lysates were denatured at 95 °C for 10 min while shaking at 1000 rpm in a thermal shaker and sonicated in a water bath for 10 min. The protein concentration of each sample was measured with a BCA protein assay kit (#23252, Thermo Scientific). 500 ng protein was used per sample and diluted with a dilution buffer containing 10% acetonitrile and 25 mM Tris-HCl, pH 8.0, to reach a 1 M GdmCl concentration. Then, proteins were digested with LysC (Roche; enzyme to protein ratio 1:50; MS-grade) while shaking at 800 rpm at 37 °C for 3.5 h. The digestion mixture was diluted again with the same dilution buffer to reach 0.5 M GdmCl, followed by tryptic digestion (Roche; enzyme to protein ratio 1:50; MS-grade) and incubation at 37 °C overnight in a thermal shaker at 800 rpm. Peptides were acidified with formic acid to a final concentration of 2%, and 250 ng of the digests were loaded onto Evotip Pure (Evosep) tips according to the manufacturer’s protocol. Peptide separation was carried out by nanoflow reverse phase liquid chromatography (Evosep One, Evosep) using the Aurora Elite column (15 cm × 75 µm ID, C18 1.7 µm beads, IonOpticks) with the 20 samples-a-day method (Whisper zoom). The LC system was coupled to a timsTOF SCP mass spectrometer (Bruker Daltonics) applying the data-independent acquisition (DIA) with parallel accumulation serial fragmentation method. MS data were processed with Dia-NN (v2.0) and searched against a library of in silico predicted human spectra. The “match between run” feature was used, and the search range was set to an *m*/*z* between 400 and 1000. The mass spectrometry data have been deposited to the ProteomeXchange Consortium (http://proteomecentral.proteomexchange.org) via the PRIDE partner repository^44^ with the dataset identifier PXD072978.

### Immunoblotting

Proteins were extracted in RIPA buffer (150 mM NaCl, 50 mM Tris, 5 mM EDTA, 1% Triton X-100, 0.25% desoxycholate, 5% sodium dodecyl sulfate (SDS)) containing protease inhibitor (Complete, Roche). 20 μg of protein per lane was separated by SDS-polyacylamide gel electrophoresis (PAGE), transferred to nitrocellulose or PVDF membrane and probed with primary antibodies. Immunoblot staining was performed for MRPL42 (rabbit anti-MRPL42; 17300-1-AP, Proteintech, 1:1000), FLAG (mouse anti-FLAG; F1804, Merck, 1:1000), OXPHOS complexes (Total OXPHOS Human WB Antibody Cocktail; ab110411; Abcam, 1:500), COX IV (rabbit anti-COX IV; #4850; Cell Signaling technology, 1:1000) and GAPDH (mouse anti-GAPDH, #AM4300, ThermoFisher, 1:20,000). Membranes were incubated with IRDye-/ horseradish peroxidase (HRP)-conjugated secondary antibodies. Signals were detected with OdysseyFc Imaging System and densitometric quantification was performed using Image Studio (LI-COR Biosciences).

### OXPHOS complex activity assay

Mitochondrial respiratory chain enzyme activities were measured at the Translational Metabolic Laboratory at Radbound University Center in patient-derived fibroblasts in a diagnostic workflow following previously described procedures^45^.

### Mitochondrial respiration

Following cell count titration experiments, 1.5 × 10^4^ cells were seeded in Seahorse XF Cell Culture Plates (Agilent) in 2 blocks with 4 wells in cell culture growth medium (see above) for 24 h, with row A and H serving as background controls. 24 h before measurement, the medium was replaced by low glucose medium (1 g/l glucose), and again 1 h before measurement by assay medium in a CO₂-free incubator. OCRs were measured on a Seahorse XFe96 Analyzer (Agilent) in real-time at 37 °C according to the Cell Mito Stress Test protocol. Basal OCR was measured for 3 cycles. Then, 3 µM oligomycin (Sigma-Aldrich) was injected for another 3 cycles to hyperpolarize the mitochondrial membrane, followed by 0.5 µM carbonyl cyanide-4-(trifluoromethoxy)phenylhydrazone (FCCP, Sigma-Aldrich) for the next 3 cycles to determine the maximal OCR and finally 0.5 µM antimycin A (Sigma-Aldrich) and 1.5 µM rotenone (Sigma-Aldrich) for another 3 cycles to inhibit the respiratory chain. Afterwards, medium was removed from cell culture plates and they were frozen at −80 °C. Protein concentration of each well was measured using a Micro BCA Protein Assay Kit (Thermo Scientific) according to the manufacturer’s instructions for normalization to protein abundance. The spare capacity (SC) was calculated as the difference between maximal and basal OCR indicating the adaptability to increased ATP demand. ATP turnover rate was calculated by subtracting proton leak from basal OCR.

### Lentiviral MRPL42 complementation

For lentiviral overexpression of MRPL42, the human *MRPL42* ORF (NM_014050.4) was labeled with a MYC-DDK tag and cloned into the lentiviral shuttle vector FUGW^46^. This lentiviral shuttle vector under the control of a CAG promoter also contained a GFP sequence and a puromycin-resistance cassette. Lentiviruses were produced in HEK293 cells by the Viral Core Facility of the Charité (vcf.charite.de). Dermal fibroblasts from the patient and an unaffected, unrelated control were transduced using lentivirus “GFP” or “MRPL42-WT” at MOI 10 in the presence of 8 µg/ml polybrene (Biotechne)^47,48^. Five days post transduction, cells were selected using 1.5 µg/ml puromycin (Sigma-Aldrich) for 48 h. The selected lentivirus-carrying cells were subsequently cultured in cell culture medium. The lentiviral production and transduction experiments were approved by the LAGeSo Berlin (89/09-34).

### Statistical analysis

Statistical analyzes were performed in GraphPad Prism 8.3 using 2way analysis of variance (ANOVA) or student’s *t*-test. Figures were designed using CoralDRAW 2020.

## Supplementary information

Supplementary Information

## Data Availability

All data needed to evaluate the conclusions in the paper are presented in the main article and in the Supplementary Data. NGS-based data have not been uploaded to a repository to ensure privacy of the affected individuals' family. The pathogenic variant has been uploaded to ClinVar (ID: 4071942). The proteomics data are available via the PRIDE partner repository with the dataset identifier PXD072978. Additional information or material may be requested from the corresponding authors.
