# Supplementary material for: A biallelic MRPL42 variant causes a combined oxidative phosphorylation deficiency syndrome revealed by multi-omics
Source: NPJ Genom Med. 2026 Apr 3;11:20. doi: 10.1038/s41525-026-00564-1 (PMC13057132; doi:10.1038/s41525-026-00564-1)
Supplement: Supplementary file 1 — Supplementary Information [file 41525_2026_564_MOESM1_ESM.pdf]

Supplemental Material: A biallelic *MRPL42* variant causes a combined oxidative phosphorylation deficiency syndrome revealed by multi-omics

Felix Boschann[1,2,*,§], Johannes Kopp[1,3,*], Susanne Römer[4], Oliver Küchler[1,5], Hristiana Lyubenova[1], Nicolai von Kügelgen[1,6], Erik Hertstein[1], Lea M. Hagelstein[1], Sebastian Brachs[7,8,9], Knut Mai[7], David Meierhofer[10], Dominik Seelow[1,5], Denise Horn[1], Markus Schülke[11,12,13], Björn Fischer-Zirnsak[1,3,13,§]

Table S1. PCR and RT-PCR primer sequence.

**Primer**	**Sequence (5’->3’)**
hMRPL42_ex2_Fwd	GCTGCAGTAAAATGGGTGATGTC
hMRPL42_ex6_Rev	GATTCCCCCAGGAACCTCCG
hMRPL42_ex2/3_Fwd	GCTGTAGCTGCAGTAAAATGGG
hMRPL42_ex2/3_Rev	TCTGGTAGAGGAGAATACGTAGA
hMRPL42_ex5/6_Fwd	TCTTTACTACTAAGCACCGTTGG
hMRPL42_ex5/6_Rev	CTTTGATTCCCCCAGGAACCTC
hGAPDH_Fwd	CTGCACCACCAACTGCTTAG
hGAPDH_Rev	ACAGTCTTCTGGGTGGCAGT

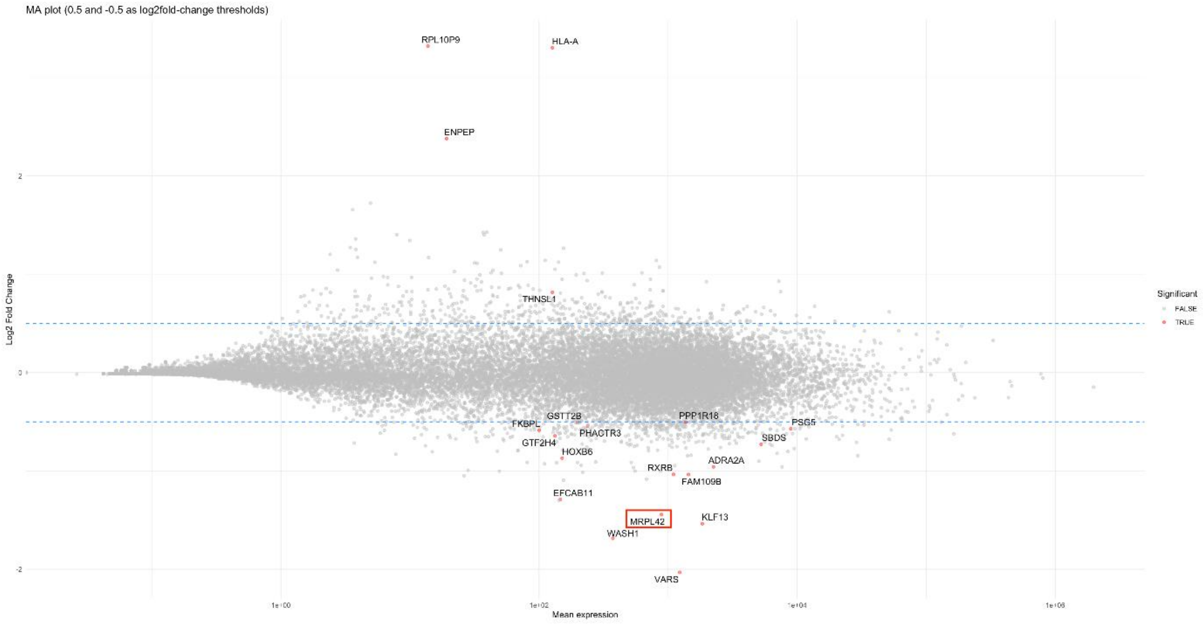

Fig. S1: MA plot displaying the estimated distribution of log2 fold changes and mean expression values from RNA-Seq data. Log2 fold changes are shrinked by the DESeq2 shrinkage estimator “normal”. Significantly differently expressed genes with a log2 fold change greater than 0.5 or less than 0.5 (thresholds are depicted as blue lines) are marked red. All differently expressed genes are labeled. *MRPL42* is marked by a red box.

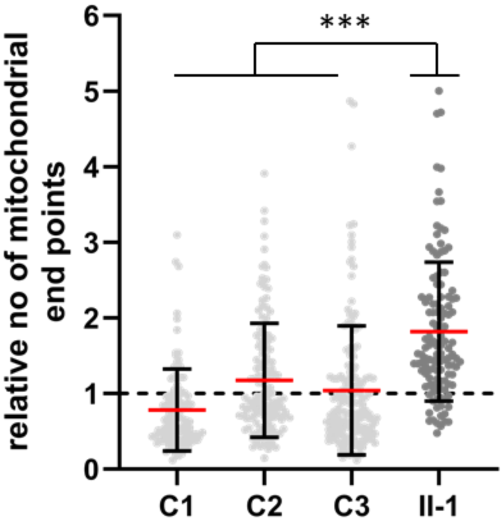

Fig. S2: Analysis of mitochondrial network fragmentation. Relative number of end points of the mitochondria network. Immunofluorescence images of patient's fibroblasts II-1 were analyzed and quantified relative to three unaffected control cell lines using ImageJ. Students t-test: P***<0.001. Diagram was generated using GraphPad Prism 8.3.

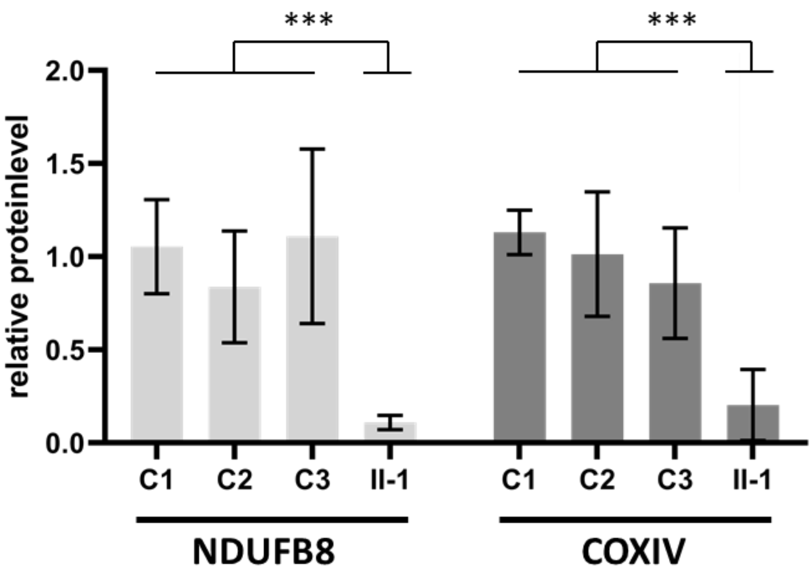

Fig. S3: Quantification of immunoblot detection of OXPHOS complex components NDUFB8 and COXIV of II-1 compared to three unaffected controls. 2way ANOVA: P***<0.001. Diagrams were generated using GraphPad Prism 8.3.

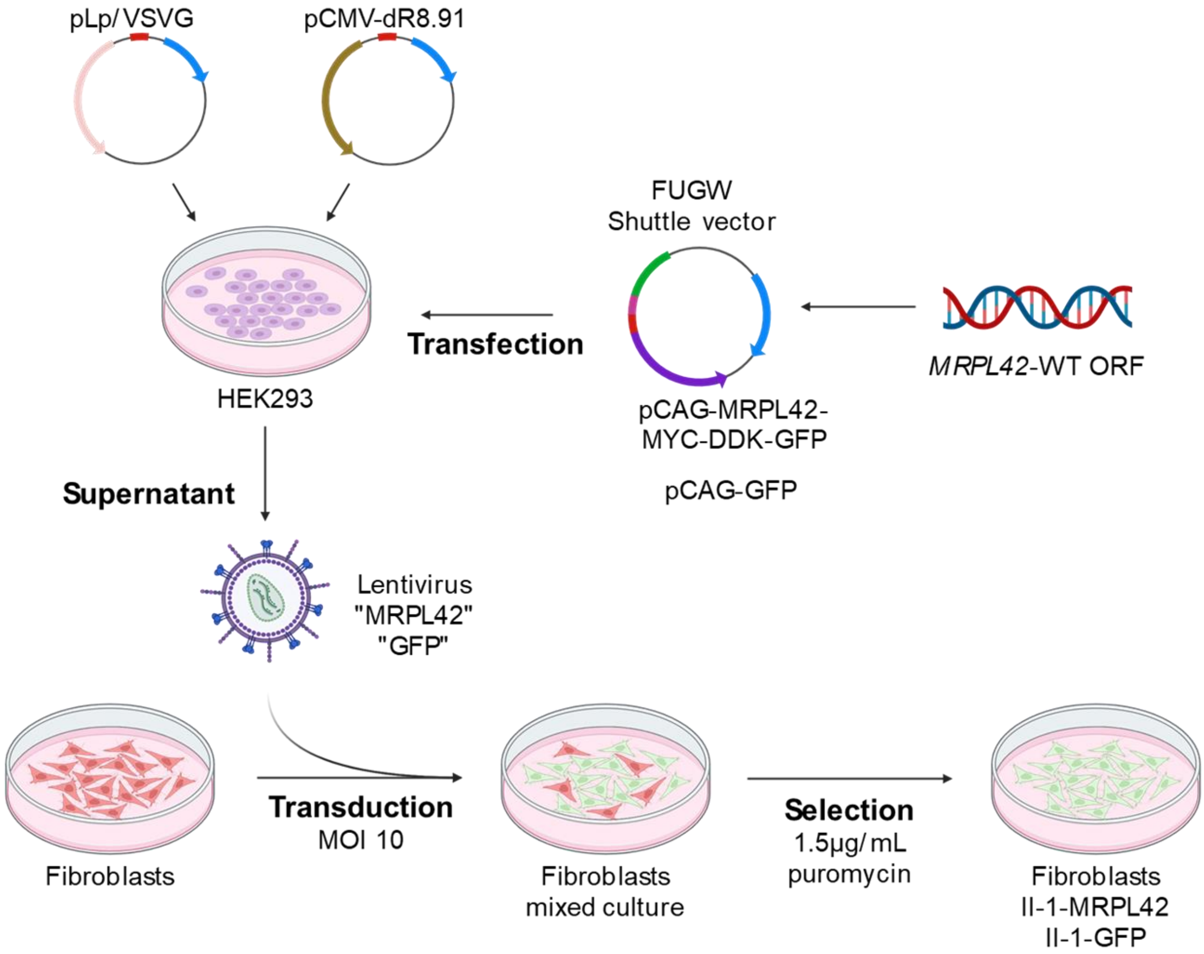

Fig. S4: Schematic illustration of lentiviral production and transduction strategy. Created in BioRender. Kopp, J. (2026) https://BioRender.com/esgtqg9 "

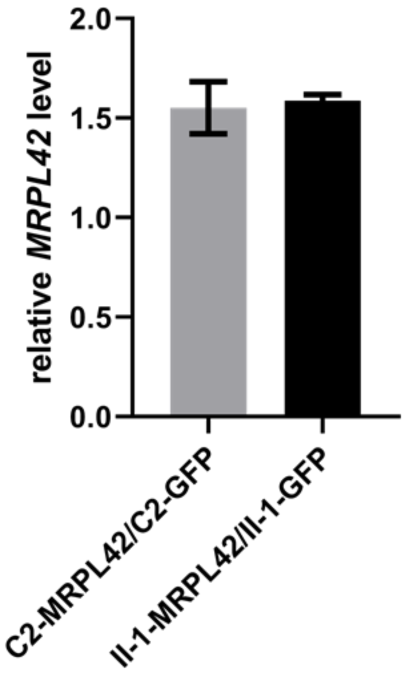

Fig. S5: Quantitative RT-PCR shows equal overexpression of MRPL42 after lentiviral gene transfer in proband’s and control fibroblasts. Diagram was generated using GraphPad Prism 8.3.

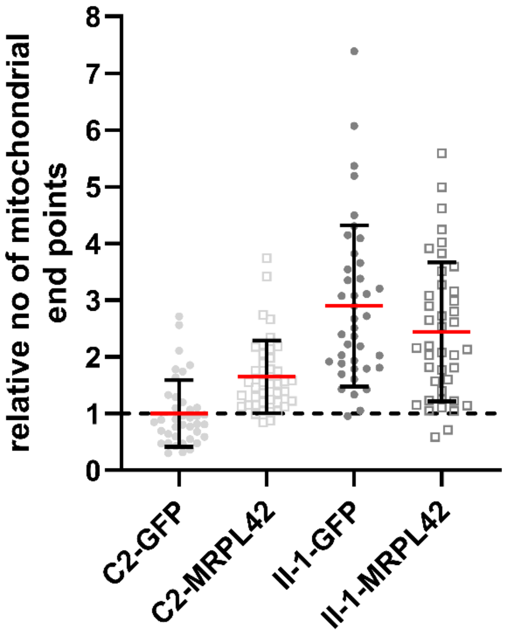

Fig. S6: Analysis of mitochondrial network fragmentation after lentiviral transduction. Relative number of junctions of mitochondria network. Immunofluorescence images were analyzed and quantified relative to C2-*GFP* using ImageJ. Diagram was generated using GraphPad Prism 8.3.

Uncroped Images from Figures 1-3

Figure 1:

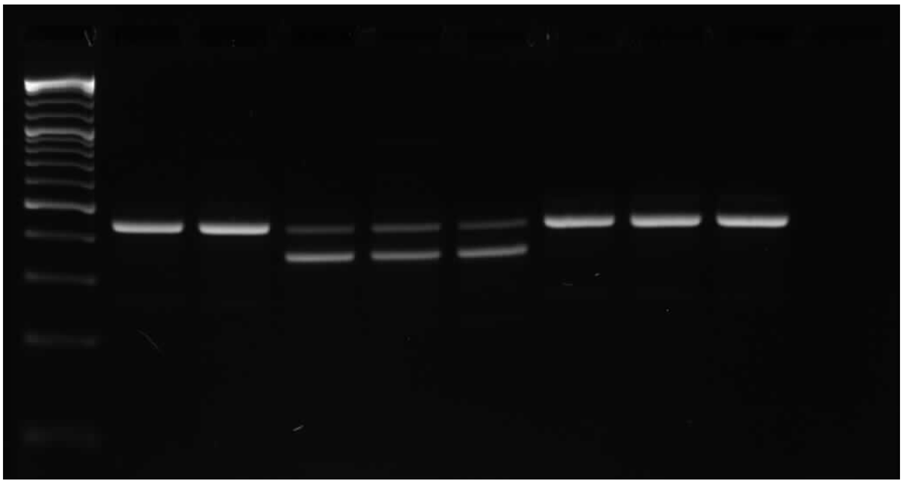

Uncroped image from Fig. 1d. *MRPL42* amplification.

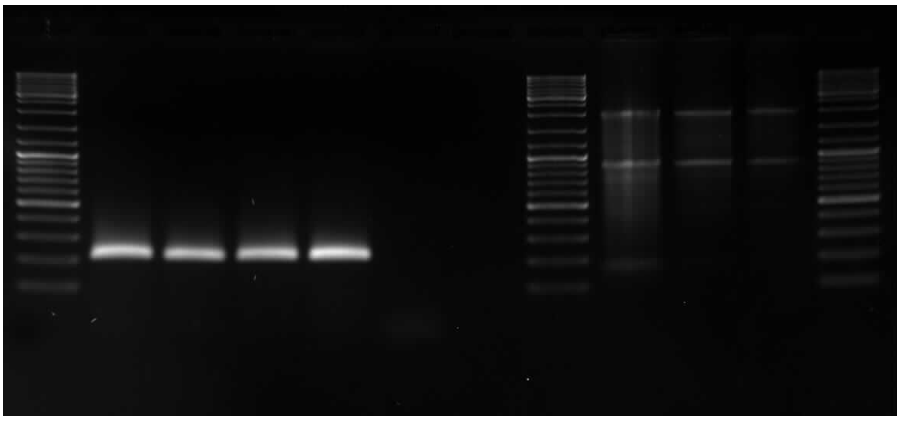

Uncroped image from Fig. 1d. *GAPDH* amplification.

Figure 2:

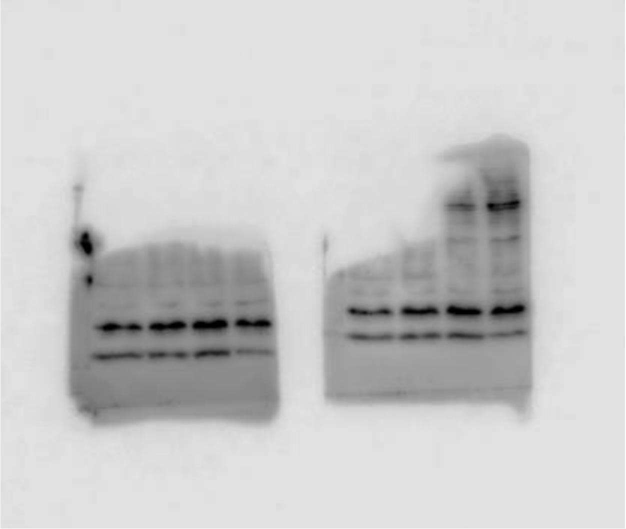

Uncroped image from Fig. 2c. MRPL42 labeling

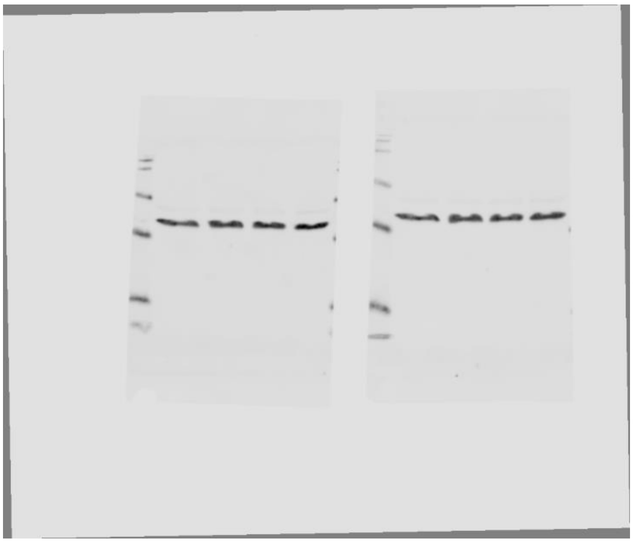

Uncroped image from Fig. 2c. GAPDH labeling

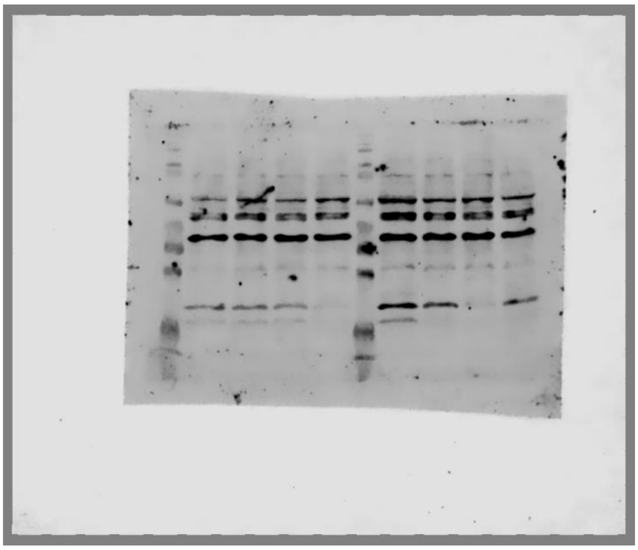

Uncroped image from Fig. 2d. OXPHOS labeling

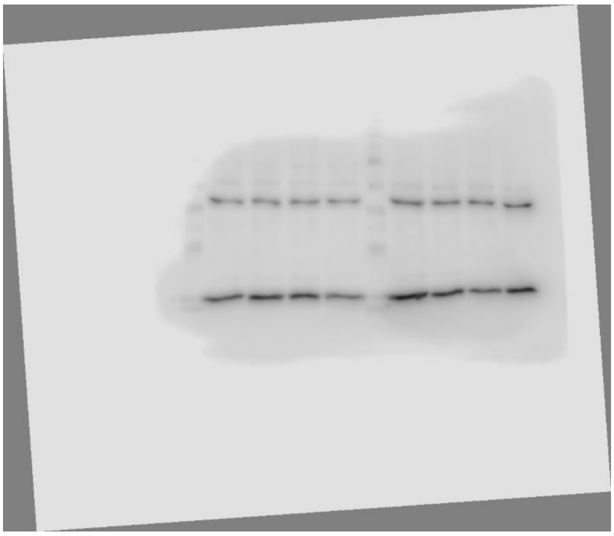

Uncroped image from Fig. 2d. COXIV labeling

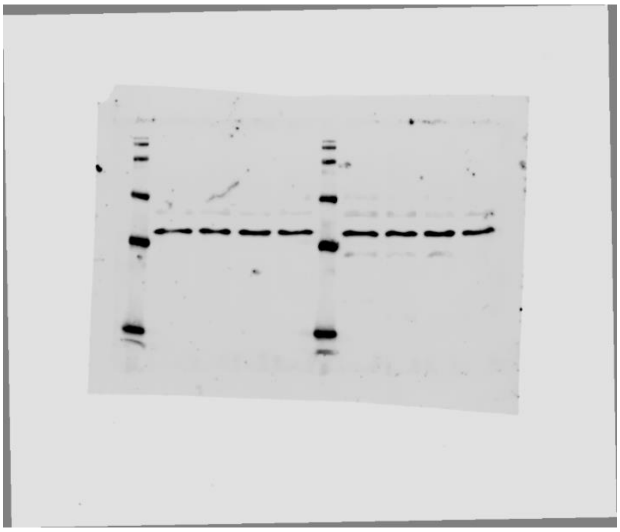

Uncroped image from Fig. 2d. GAPDH labeling

Figure 3:

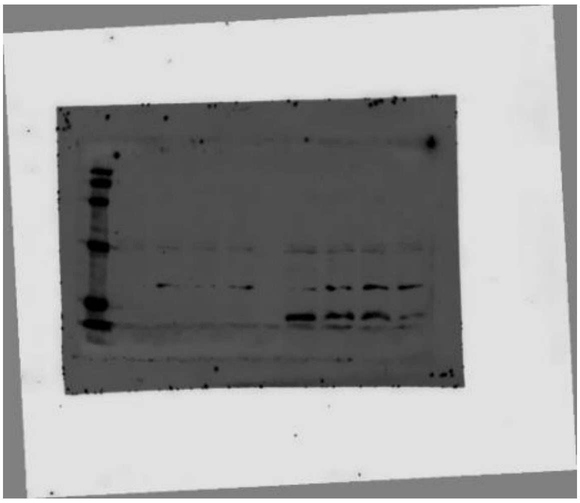

Uncroped image from Fig. 3b. GFP labeling

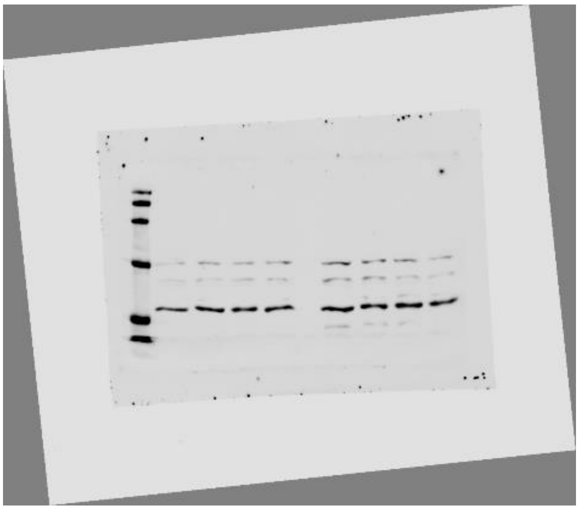

Uncroped image from Fig. 3b. GAPDH labeling

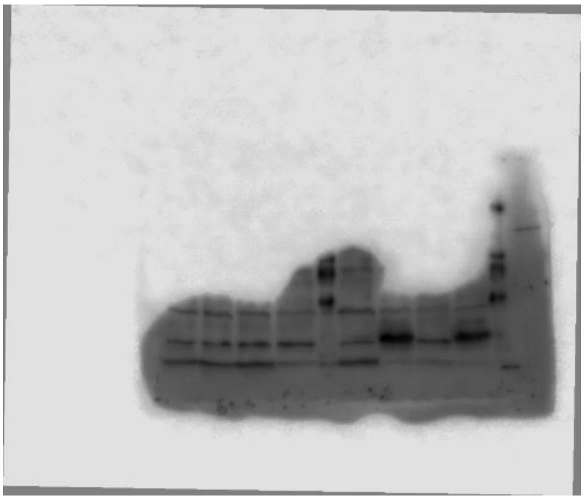

Uncroped image from Fig. 3b. MRPL42 labeling

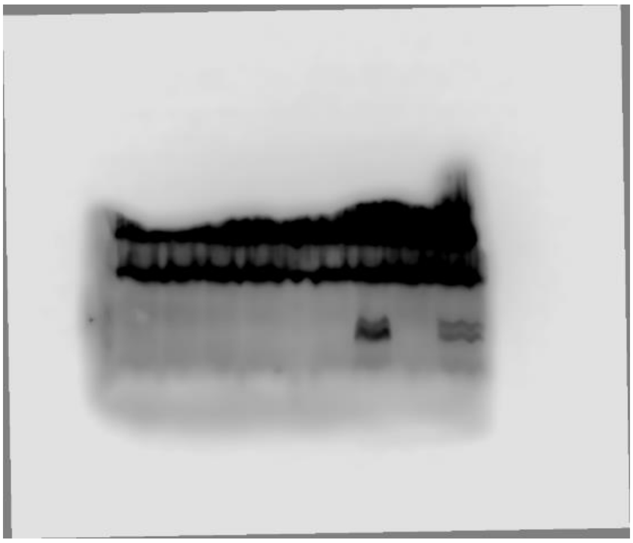

Uncroped image from Fig. 3b. FLAG labeling

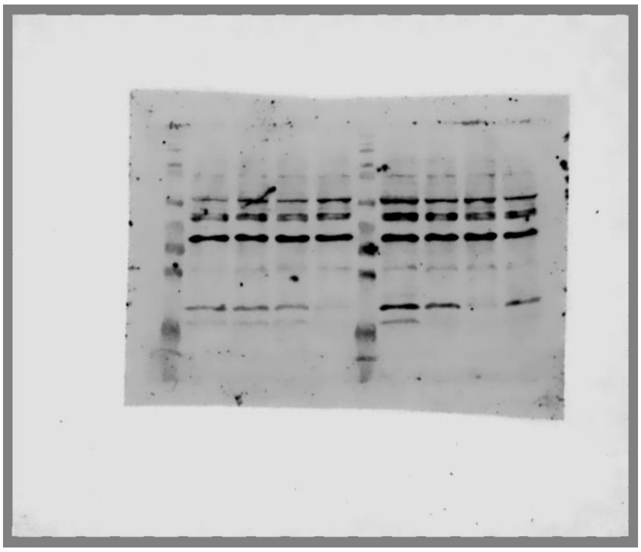

Uncroped image from Fig. 3c. OXPHOS labeling

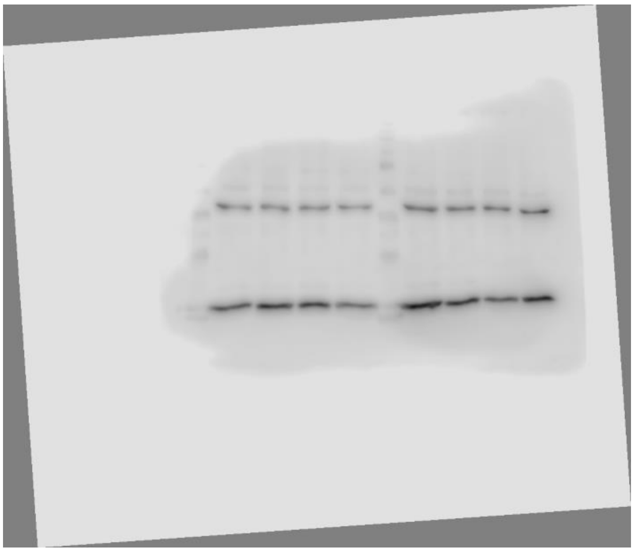

Uncroped image from Fig. 3c. COXIV labeling

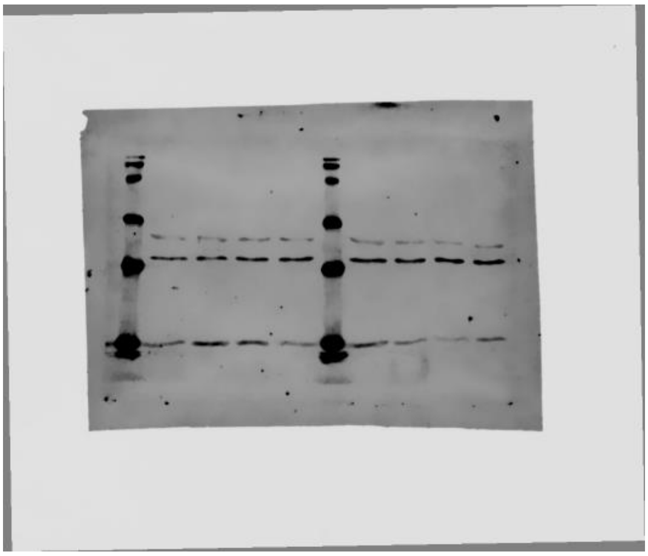

Uncroped image from Fig. 3c. GAPDH labeling
